# Functional microbial diversity explains groundwater chemistry in a pristine aquifer

**DOI:** 10.1186/1471-2180-13-146

**Published:** 2013-06-24

**Authors:** Theodore M Flynn, Robert A Sanford, Hodon Ryu, Craig M Bethke, Audrey D Levine, Nicholas J Ashbolt, Jorge W Santo Domingo

**Affiliations:** 1 Biosciences Division, Argonne National Laboratory, Argonne, IL 60439, USA; 2 Department of Geology, University of Illinois at Urbana-Champaign, Urbana, IL 60616, USA; 3 Office of Research and Development, U.S. Environmental Protection Agency, Cincinnati, OH 45248, USA; 4 Battelle, Washington, DC, 20024, USA

## Abstract

**Background:**

The diverse microbial populations that inhabit pristine aquifers are known to catalyze critical in situ biogeochemical reactions, yet little is known about how the structure and diversity of this subsurface community correlates with and impacts upon groundwater chemistry. Herein we examine 8,786 bacterial and 8,166 archaeal 16S rRNA gene sequences from an array of monitoring wells in the Mahomet aquifer of east-central Illinois. Using multivariate statistical analyses we provide a comparative analysis of the relationship between groundwater chemistry and the microbial communities attached to aquifer sediment along with those suspended in groundwater.

**Results:**

Statistical analyses of 16S rRNA gene sequences showed a clear distinction between attached and suspended communities; with iron-reducing bacteria far more abundant in attached samples than suspended, while archaeal clones related to groups associated with anaerobic methane oxidation and deep subsurface gold mines (ANME-2D and SAGMEG-1, respectively) distinguished the suspended community from the attached. Within the attached bacterial community, cloned sequences most closely related to the sulfate-reducing *Desulfobacter* and *Desulfobulbus* genera represented 20% of the bacterial community in wells where the concentration of sulfate in groundwater was high (> 0.2 mM), compared to only 3% in wells with less sulfate. Sequences related to the genus *Geobacter*, a genus containing ferric-iron reducers, were of nearly equal abundance (15%) to the sulfate reducers under high sulfate conditions, however their relative abundance increased to 34% when sulfate concentrations were < 0.03 mM. Also, in areas where sulfate concentrations were <0.03 mM, archaeal 16S rRNA gene sequences similar to those found in methanogens such as *Methanosarcina* and *Methanosaeta* comprised 73–80% of the community, and dissolved CH_4_ ranged between 220 and 1240 μM in these groundwaters. In contrast, methanogens (and their product, CH_4_) were nearly absent in samples collected from groundwater samples with > 0.2 mM sulfate. In the suspended fraction of wells where the concentration of sulfate was between 0.03 and 0.2 mM, the archaeal community was dominated by sequences most closely related to the ANME-2D, a group of archaea known for anaerobically oxidizing methane. Based on available energy (∆G_A_) estimations, results varied little for both sulfate reduction and methanogenesis throughout all wells studied, but could favor anaerobic oxidation of methane (AOM) in wells containing minimal sulfate and dihydrogen, suggesting AOM coupled with H_2_-oxidizing organisms such as sulfate or iron reducers could be an important pathway occurring in the Mahomet aquifer.

**Conclusions:**

Overall, the results show several distinct factors control the composition of microbial communities in the Mahomet aquifer. Bacteria that respire insoluble substrates such as iron oxides, i.e. *Geobacter*, comprise a greater abundance of the attached community than the suspended regardless of groundwater chemistry. Differences in community structure driven by the concentration of sulfate point to a clear link between the availability of substrate and the abundance of certain functional groups, particularly iron reducers, sulfate reducers, methanogens, and methanotrophs. Integrating both geochemical and microbiological observations suggest that the relationships between these functional groups could be driven in part by mutualism, especially between ferric-iron and sulfate reducers.

## Background

Microbial life thrives in natural waters, including those found deep in the terrestrial subsurface [1]. Groundwater there may contain little or no dissolved oxygen, and in such cases microbial activity is dominated by populations that can respire using other electron acceptors such as ferric iron, sulfate, or carbon dioxide. By catalyzing a diverse array of oxidation and reduction reactions, microorganisms (i.e., bacteria and archaea) exert strong influence over the chemistry of groundwater [2], thereby controlling the rates of mineral weathering in aquifers [3], the fate and transport of metals and organic compounds [4], and the geological sequestration of greenhouse gases [5]. Knowledge of the activity and composition of groundwater microbial communities across different spatial scales is therefore critical to the understanding of subsurface biogeochemistry.

Rather than being segregated into distinct zones where a single functional group predominates, molecular analyses commonly show diverse microbial populations coexisting in aquifers, regardless of how the bulk groundwater is classified by geochemical criteria. For example, molecular studies in an aquifer near Cerro Negro (New Mexico, U.S.) have demonstrated the presence of sulfate-reducing, iron-reducing, and denitrifying bacteria in groundwater systems where geochemical indicators point to sulfate reduction alone as the predominant form of respiration [6-9].

Currently there is limited knowledge of how microbial diversity relates to biogeochemical processes on an ecosystem scale [10]. Studies of microbial ecology in aquifers are frequently confined to specific taxa of interest, such as groups known to degrade a particular contaminant or to comparisons of pristine and contaminated areas [4,11]. Furthermore, most molecular characterizations of aquifer ecosystems have focused on microbiota suspended in pumped groundwater, which at least partially ignores the microbial fraction attached to sediment particles [12,13]. While it is known that attached populations constitute the majority of cells in the subsurface and there are physiological differences between attached and suspended microbial communities, few studies have examined differences between these two fractions [14,15]. One such difference associated with a specific group involves the iron-reducing bacteria, which are usually associated with a solid substrate [16] and therefore are expected to be underrepresented in the bulk groundwater.

The Mahomet aquifer in east-central Illinois hosts distinct zones of high and low sulfate groundwater [17]. This aquifer contains a diverse community of iron-reducing and sulfate-reducing bacteria in which sulfate has been proposed as a key discriminant of bacterial community structure [18]. Specifically, in high sulfate wells, sulfate reducers have been shown to co-exist with iron reducers throughout the aquifer [18], contrary to previous notions that sulfate reduction is excluded under iron-reducing conditions [19-21]. Previous studies focused exclusively on bacterial populations, leaving the distribution of archaeal populations such as methanogens unexplored. Dissolved methane exists at significant concentrations in this aquifer and isotopic studies indicate that it is of microbial origin [22], suggesting methanogenesis has occurred in the Mahomet aquifer alongside iron reduction and sulfate reduction. In this study, we examined in greater detail than previous studies the community composition of bacteria and archaea in both groundwater and Mahomet aquifer sediment using partial-length 16S rRNA gene sequence analysis. Furthermore, we calculate the available thermodynamic energy for microbial respiration and compare this available energy with the distribution of phylotypes with which a particular mode of respiration is associated.

## Methods

### Sample collection

**Figure 1:**
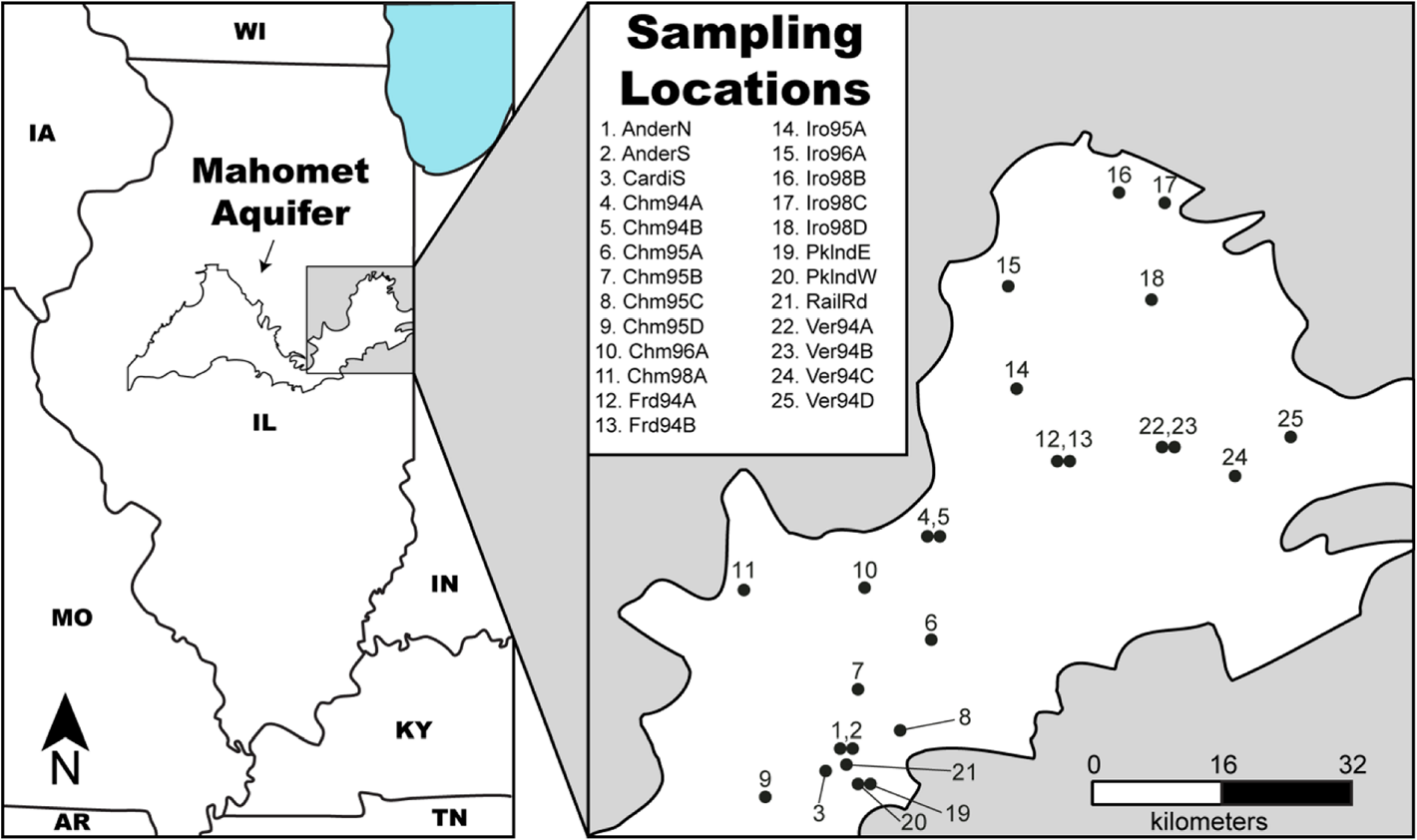
Map of the east-central Illinois region of the Mahomet aquifer showing the location of the wells sampled in this study.

Samples for geochemical and microbiological analysis were collected from 25 observation wells located in the east-central Illinois region of the Mahomet (Figure 1). These wells draw groundwater from one of two sedimentary horizons, the younger, shallower Glasford formation or the older, deeper Banner formation. Wells were screened at the bottom of the respective formation over a span of 1.5 m at depths ranging from 41 m to 117 m below ground surface. These formations are comprised of unconsolidated sands and gravels that were deposited as glacial outwash during the Pleistocene era and are interbedded with confining layers of glacial till that serve as aquitards [23]. The bedrock underlying the north-central part of our sampling area is composed of pyritic coal and shale, whereas bedrock to the south and east is largely carbonate [17]. Locally, saline groundwater from the coal and shale passes upward and mixes with the dilute, meteoric groundwater of the shallower aquifers. Groundwater in this area of the Mahomet contains little modern recharge and no evidence exists of any anthropogenic contaminants [22].

Before filtering suspended cells from groundwater or deploying in situ “traps” of sterilized sediment to sample attached microbes, stagnant water was pumped out of the well at a rate of 8 L min^-1^ using a Grundfos^®^ Redi-Flo II electric submersible pump. During pumping, the pH, temperature and electrical conductivity were monitored using an Oakton pH/CON 300 Meter (Oakton Instruments, Vernon Hills, IL) and recorded at three minute intervals. No samples were taken until readings for all three parameters stabilized for three consecutive measurements. All groundwater samples for geochemical analyses were filtered in the field using a 0.2 μm pore size Supor-200^®^ polyethersulfone membrane (Pall Life Sciences). For analysis of dissolved inorganic carbon (DIC), 3 mL of groundwater was collected using a degassed syringe, then injected into a stoppered 70 mL serum bottle previously purged using O_2_-free, ultrapure N_2_ gas and 2 g of crystalline phosphoric acid (H_3_PO_4_). Samples for dissolved organic carbon (DOC) analyses were stored in amber glass bottles and preserved using sulfuric acid (0.5% v/v). Samples were stored on water ice in a sealed cooler for transport to the lab and kept refrigerated until they could be analyzed.

Microbial cells suspended in groundwater were filtered from two liters of groundwater using a 90 mm Supor-200^®^ filtration membrane. After collecting duplicate filters and immediately freezing them on dry-ice, an in situ sediment trap containing sterilized aquifer solids (see Additional file 1) was lowered into each well where it could be colonized by native microbes [15]. Sediment traps were lowered to the depth of the screened interval of each well and retrieved after 98 to 137 days of incubation, allowing active microbial populations to colonize the initially-sterile solids [24]. Upon retrieval, sediment samples were immediately placed into separate sterile Whirl-Pak^®^ bags and stored in coolers filled with dry ice. All microbiological samples (filters and sediments) were transported to the laboratory within four hours whereupon they were transferred to a -80°C freezer and stored awaiting further analysis.

Aqueous concentrations of methane and hydrogen in groundwater were determined using passive diffusion sampling [25]. In situ gas samplers were equilibrated in an individual well for at least one week and then retrieved. Triplicate samples of dissolved gases were immediately injected into stoppered, N_2_-purged serum bottles for storage. The concentrations of major anions (F^–^, Cl^–^, Br^–^, NO_3_^–^, PO_4_^3–^, SO_4_^2–^) in groundwater samples were measured using a Metrohm Advanced ion chromatograph with a detection limit of 10 μM (Metrohm USA, Houston, TX). DOC analyses were performed at the Illinois Sustainable Technology Center using a Shimadzu TOC-VCPN carbon analyzer with a detection limit of 0.4 mg kg^–1^.

Methane and DIC concentrations were measured using an SRI 8610 gas chromatograph (SRI International, Menlo Park, CA) coupled to a thermal conductivity detector (TCD) and a flame ionization detector (FID). TCD measurements were used to determine DIC and dissolved methane concentrations greater than >100 μM, while the FID was used to measure methane <100 μM. Hydrogen concentrations were determined using the same GC equipped with a reducing gas detector (RGD). The RGD detector produced reliable concentration measurements down to 0.5 nM. Gas phase concentrations of CO_2_, methane and hydrogen within the passive diffusion samplers were converted to aqueous phase concentrations using the temperature-corrected Ostwald coefficient [26], taking into account the total dissolved gas pressure in the system as measured using a Hydrolab MiniSonde 4a^®^ (Hach Hydromet, Loveland, CO).

### Energy available for microbial respiration

The thermodynamic energy available (∆*G*_A_) to particular functional groups of microbes through respiration was calculated according to the equation:

(1) $$∆G_{r}=∆G^{{}^{\circ}}{}_{T}+\mathrm{RT}ln\prod_{i}{\left(y_{i}\times m_{i}\right)}^{v_{i}}$$

Where ∆G^°^_T_ is the standard state free energy change at temperature *T* (K), *R* is the universal gas constant, and *y*_*i*_, *m*_*i*_*,* and *v*_*i*_ are the activity coefficients, molal concentrations, and reaction coefficients of the species involved in the redox reaction. The ∆*G*_A_ for a particular functional group of microbes is equal to the amount of free energy released by that group’s respiratory reaction (∆*G*_r_). The amount ∆*G*_r_ is equal to the sum of the electron donating (∆*G*_don_) and electron accepting (∆*G*_acc_) half-reactions, and the available energy (∆*G*_A_) is therefore the inverse of the energy released (-∆*G*_r_) [27]. The energy available from electron donating and accepting half-reactions was calculated in The Geochemist’s Workbench^®^ using the “thermo.dat” database of thermodynamic data compiled by Lawrence Livermore National Laboratory [28]. Activity coefficients (*y*_*i*_) were calculated from the overall chemical composition of the groundwater using the extended Debye-Hückel equation [29].

### Molecular assays and sequence analyses

Total DNA was extracted from each sediment trap and each filter membrane collected from the wells following the method of Tsai and Olson [30] with some minor modifications (see Additional file 1). DNA extracts were used to amplify 16S rRNA genes using bacterial (i.e., 8 F and 787R) and archaeal (i.e., 25 F and 958R)-specific primers (see Additional file 1). Amplification products were cloned into pCR4.1 TOPO TA vector following the manufacturer’s instructions (Invitrogen™, Carlsbad, CA). Clones were sequenced using the BigDye^®^ Terminator sequencing chemistry (Applied Biosystems, Foster City, CA) as described elsewhere [31]. A minimum of 192 clones per sample were processed in this study. Raw sequence data was checked for quality and assembled into contigs using Sequencher^®^ v4.10.1 (Gene Codes Corp, Ann Arbor, MI), and then screened for chimeras using Bellerophon [32]. For the phylogenetic analyses bacterial and archaeal sequences were aligned using the algorithm implemented in the program Mothur [33] against a high-quality reference alignment selected from the Greengenes 16S rRNA gene database [34]. Unique, chimera-free reference sequences were chosen from the 12 October 2010 release of Greengenes using ARB [35]. Cloned sequences from the Mahomet that aligned poorly to the reference database or contained ambiguous base calls were discarded. The phylogeny of archaeal and bacterial 16S rRNA gene sequences was classified in Mothur using the “Hugenholtz” taxonomic nomenclature in Greengenes [34]. Phylogenetic trees were constructed in ARB by adding cloned sequences to the Greengenes reference tree [36] using the ARB parsimony algorithm [35].

The community richness of bacteria and archaea in the Mahomet was estimated using Mothur [33]. 16S rRNA gene sequences were clustered into operational taxonomic units (OTUs) based on an average nucleotide similarity at fixed cutoffs. Sequences with an average nucleotide similarity of 97% were binned together into a single OTU. The similarity of individual communities of bacterial and archaeal members across the Mahomet was quantified using the Bray-Curtis coefficient [37]. Archaeal and bacterial communities were grouped together for these analyses on the basis of sample type (attached or suspended) and geochemical zone [15,17,18].

Bacterial and archaeal community composition differences were quantified by calculating the Bray-Curtis similarity coefficient and using multidimensional scaling (MDS) to create a two dimensional visualization of the extent to which communities differ from one another. MDS graphs were plotted using a non-metric configuration in which the distance between any two points is inversely proportional to their similarity. All MDS analyses were performed using the Primer-6 software package (Primer-E Ltd., Plymouth, UK). The overall similarity of the bacterial and archaeal communities within groups of wells was calculated using the analysis of similarity (ANOSIM) [38]. Specifically, *R*-values (R_ANOSIM_) were used to establish the dissimilarity of different paired-groups of microbial communities (e.g. communities from no sulfate vs. high sulfate groundwater). R_ANOSIM_ > 0.75 indicate two microbial communities (i.e. the attached and suspended communities from various wells in an aquifer) have characteristic structures largely distinct from one another [39]. A value of R_ANOSIM_ between 0.25 and 0.75 indicates communities within each group cluster separately from those in the other, with some overlap, while an R_ANOSIM_ < 0.25 indicates communities in one group are almost indistinguishable from those in the other. SIMPER (similarity percentage) was used to calculate the extent to which individual OTUs contribute to the dissimilarity groups sets and to rank the populations from most to least responsible for the differences between groups [40,41]. Representative sequences from each OTU were identified using Mothur and identified using the Greengenes reference taxonomy as described above. Representative sequences were deposited in GenBank under accession numbers KC604413 to KC604575 and KC604576 to KC607489.

## Results

### Groundwater geochemistry

**Figure 2:**
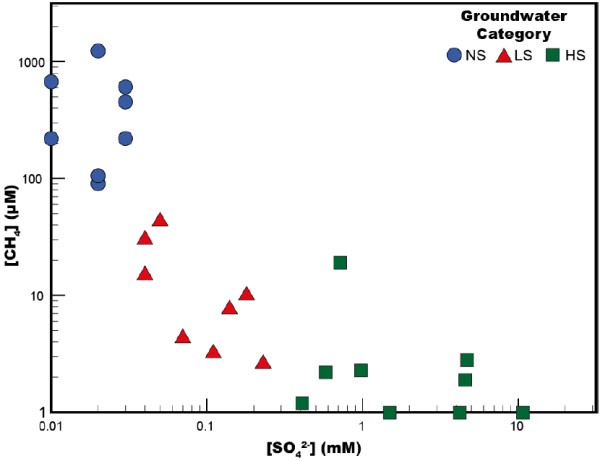
**A comparison of the methane (CH**_**4**_**) and sulfate (SO**_**4**_^**2-**^**) concentrations of individual wells in the Mahomet aquifer.** The amount of sulfate in HS wells is > 0.2 mM, is between 0.03 and 0.2 mM in LS wells, and is less than 0.03 mM in NS wells.

Table 1 shows that the concentrations of sulfate (SO_4_^2–^), methane (CH_4_), and dihydrogen (H_2_) in groundwater from the Mahomet wells each varied over several orders of magnitude (Table 1). The concentration of sulfate ranged from 10.7 mM to below the detection limit of 0.01 mM. We used the sulfate concentration in groundwater samples to classify each well following the scheme devised by Panno *et al.*[17] for the Mahomet aquifer. We designated nine wells as high sulfate (HS; [SO_4_^2-^] > 0.2 mM), eight as low sulfate (LS; [SO_4_^2-^] = 0.03 - 0.2 mM), and eight wells as negligible sulfate (NS, [SO_4_^2-^] < 0.03 mM). While methane was not considered in Panno *et al.* classification, we found an inverse relationship exists between the concentration of dissolved methane and that of sulfate (Figure 2). Dissolved methane ranged from below detection (< 0.2 μM) to 1240 μM, with the highest concentrations occurring in NS wells ([CH_4 (aq)_] = 220–1240 μM). Dissolved methane was not detected in three of the eight HS wells, and concentrations were < 3 μM in four of the others. The concentration of dissolved H_2_, however, ranged from 3 to 240 nM and did not correlate to any other measured geochemical species.

**Table 1:** Geochemistry of groundwater in Mahomet aquifer wells

**Well**	**Temp. (°C)**	**pH**	**sp. Cond. (μS.cm**^**-1**^**)**	**[SO**_**4**_^**2-**^**] (mM)**^**a**^	**[CH**_**4**_**]**_**aq **_**(μM)**^**b**^	**[H**_**2**_**]**_**aq **_**(nM)**^**c**^	**DIC (mM)**^**d**^	**DOC (mg.L**^**-1**^**)**^**e**^
*High sulfate (HS) wells*								
Chm94B	13.7	7.5	707	0.58	< 0.2	25	7.8	2.2
Chm96A	13.8	7.5	663	0.41	1	3	7.2	1.3
Frd94A	14.2	7.5	760	0.98	2	3	7.4	< 0.4
Iro95A	14.3	7.5	943	1.50	1	60	n/a	3.3
Iro96A	12.1	7.5	1254	4.23	1	n/a	n/a	n/a
Iro98B	13.0	7.6	1277	4.68	3	10	6.6	43.0
Iro98D	13.6	7.8	759	0.72	19	180	7.9	1.9
Ver94A	14.4	7.5	1279	4.57	2	n/a	6.7	1.8
Ver94B	13.7	7.3	1893	10.73	1	89	4.8	1.1
*Low sulfate (LS) wells*								
Chm94A	14.1	7.6	651	0.07	4	n/a	8.0	3.6
Chm95A	14.0	7.6	649	0.14	8	4	7.7	2.1
Chm95B	13.8	7.9	670	0.04	30	3	7.9	2.0
Chm95C	13.7	7.7	601	0.11	3	20	6.6	0.5
Frd94B	15.4	7.6	611	0.05	43	9	7.4	< 0.4
Iro98C	13.3	7.4	664	0.04	15	66	7.6	2.3
Ver94C	13.6	7.7	616	0.23	3	46	7.4	1.1
Ver94D	13.9	7.7	621	0.18	10	n/a	7.7	0.8
*Negligible sulfate (NS) wells*								
AnderN	14.8	7.6	617	0.02	91	144	6.6	n/a
AnderS	15.1	7.1	860	0.02	1237	175	25.9	n/a
CardiS	13.6	7.7	645	0.03	454	240	7.5	n/a
Chm95D	14.0	7.8	625	< 0.01	220	12	7.6	1.6
Chm98A	13.7	7.7	714	< 0.01	676	24	7.9	4.2
PklndE	14.6	7.6	678	0.03	221	63	8.7	n/a
PklndW	14.4	7.5	725	0.03	611	100	6.0	n/a
RailRd	14.4	7.7	661	0.02	106	50	6.4	n/a

^a^ The detection limit for sulfate is 0.01 mM.

^b^ The detection limit for dissolved methane is 0.2 μM.

^c^ The detection limit for dissolved hydrogen is 0.5 nM.

^d^ The detection limit for dissolved inorganic carbon is 0.5 mM.

^e^ The detection limit for dissolved organic carbon is 0.4 mg L^–1^.

### Available energy

We evaluated the available energy (∆G_A_), which is equal to the –∆G_r_[42], for different metabolisms using H_2_ as an electron donor based on the geochemical data shown in Table 1. In contrast to what might be expected from previous work [43,44], H_2_ concentrations did not increase as methanogenic conditions became predominant in the NS wells and therefore had little impact on the available energy calculation. Thus the ∆*G*_A_ varied little for both sulfate reduction and methanogenesis throughout all wells, despite changes in the concentration of key chemical species sulfate and methane (Additional file 1: Table S1). Based on the concentrations of the substrates involved, sulfate reduction via the oxidation of H_2_ yielded on average calculated ∆*G*_A_ values between 43 and 102 kJ mol^-1^, while methanogenesis yielded ∆*G*_A_ values between 17 and 56 kJ mol^-1^ in all the wells sampled. In contrast to these energetically favored reactions, anaerobic oxidation of methane (AOM), which proceeds via a reversal of the methanogenic pathway [45], was not energetically favorable (∆*G*_A_ < 0) at the bulk concentrations of dihydrogen (H_2_) measured in the Mahomet aquifer groundwater. Since bulk H_2_ concentrations may differ from local sites in the aquifer we calculated the threshold concentration where AOM would yield a positive ∆G_A_ and found it to be less than 0.2 nM (Additional file 1: Figure S3). It is therefore possible that, if coupled with H_2_-oxidizing organisms such as sulfate reducers or iron reducers, AOM could occur in LS wells, where 16S rRNA sequences most closely related to archaea capable of anaerobically oxidizing methane predominate (see below). The direct coupling of methane oxidation to sulfate reduction by a single organism where H_2_ is not an intermediate would also yield a positive ∆G_A_ in the samples collected (Additional file 1: Table S1).

### Microbial composition and diversity analysis

A total of 16,952 clones (8,786 bacteria, 8,166 archaea) were sequenced. Chimeric sequences detected by Bellerophon represented less than 3% of all sequences and were discarded before any further analyses were performed. At a sequence similarity cutoff of 97%, the bacterial community contains 2,681 unique operational taxonomic units (OTUs). Collectors curves showed how the observed richness increased with greater sequencing depth, indicating that the total richness of Mahomet bacterial community is likely to be even greater than quantified here (Additional file 1: Figure S1). Archaeal sequence diversity showed one order of magnitude less OTU richness than their bacterial counterparts, containing 271 unique OTUs. In contrast with the bacterial sequences, the collectors curves indicated that our depth of sequencing accounted for most of the richness of the archaeal community attached to the sediment samplers, but suggested the suspended archaea were undersampled in groundwater (Additional file 1: Figure S2). This may be due to insufficient sediment exposure time to the archaeal community or reflects a preference for most archaea to remain suspended in the groundwater.

### Comparison of attached and suspended communities

We separately examined the microbial communities in each well, and quantified how the bacteria and archaea attached to our in situ samplers differed from those suspended in groundwater. These assemblages of microbial communities are hereafter referred to as ATT and SUS, respectively. The 5,620 sequences analyzed from ATT bacterial communities contained 2,072 OTUs at the 97% sequence similarity cutoff, compared to 1,216 OTUs identified among the 2,585 sequences in the SUS fraction (Table 2). We analyzed a random set of 2,585 ATT sequences to see if the greater richness in the ATT community was simply a result of greater sequencing depth, and found this normalized subset contained only 1,243 OTUs, which is nearly identical to the number of OTUs identified for the SUS samples. Although only 152 OTUs were detected in both ATT and SUS groups, these accounted for 37% and 31% of the sequences, respectively, indicating these shared populations made up significant fractions of both communities.

**Table 2:** Unique and shared richness of microbial communities

	***Bacteria***		***Archaea***	
	**ATT**^**a**^	**SUS**^**b**^	**ATT**^**a**^	**SUS**^**b**^
Total OTUs^c^	2,072	1,216	60	266
Normalized OTUs^d^	1,243	–	55	–
% OTUs shared	12%	13%	31%	6%
% Sequences in Shared OTUs	37%	31%	90%	22%

^a^ ATT = Microbial communities attached to in situ samplers.

^b^ SUS = Microbial communities suspended in groundwater.

^c^ Operational taxonomic units (OTUs) calculated using a cutoff of 97% average nucleotide similarity.

^d^ The number of OTUs found in a randomized subset of *n* sequences where = the number of suspended samples. This was done to account for the greater number of ATT sequences among both bacteria and archaea.

The archaeal community was considerably less diverse than the bacterial community, even though we analyzed a comparable number of sequences. The 4,870 archaeal sequences analyzed from ATT samples contained 60 total OTUs, while the 3,143 sequences from SUS archaea contained 266 OTUs. Seventeen OTUs were observed in both ATT and SUS archaeal fractions and 90% of ATT archaeal sequences fell within the shared OTUs, compared to only 22% of SUS archaea (Table 2).

**Figure 3:**
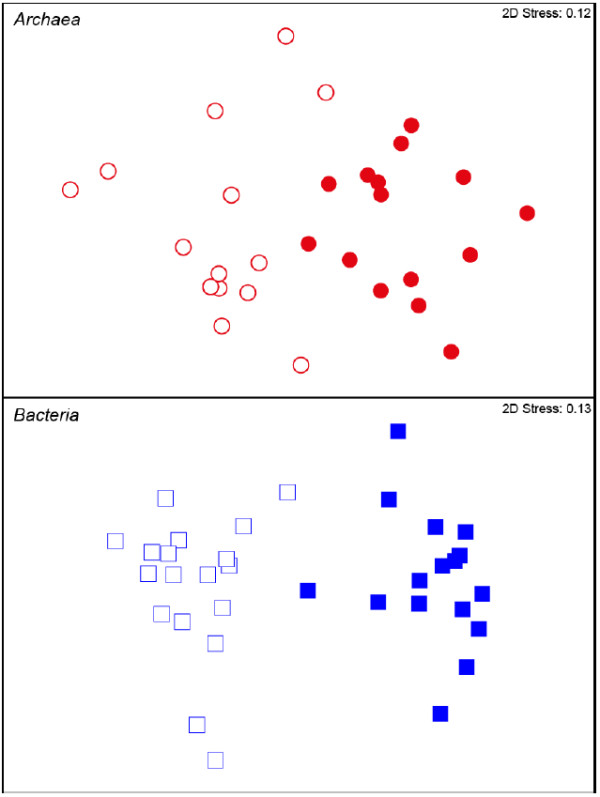
**Nonmetric multidimensional scaling (MDS) ordination of the Bray-Curtis similarity coefficient for communities of archaea and bacteria in the Mahomet aquifer.** Attached samples (filled markers) are of microbes that colonized in situ sampler sediment while suspended samples (open markers) were filtered from groundwater as it was pumped from the aquifer. For MDS analysis, sequences across all communities with 97% or greater sequence similarity were binned into operational taxonomic units (OTUs). The stress indicated in the upper right corner is the amount of strain imposed on the ordination when fitting it into two dimensions.

To quantify the difference in composition between ATT and SUS bacterial and archaeal communities we used a variety of multivariate statistical tools including analysis of similarity (ANOSIM), nonmetric multidimensional scaling (MDS), and similarity percentage (SIMPER). To avoid biasing results we chose sequences only from wells where both ATT and SUS samples were available. Using 97%-similarity OTUs, we calculated an R_ANOSIM_ of 0.915 for bacteria and 0.508 for archaea (*p* < 0.001%), indicating that each habitat type contained a microbial community with a distinct composition [39]. MDS plots of bacterial and archaeal community relatedness in the Mahomet aquifer mirror the results of ANOSIM as they show that communities that attach to the sediment traps differed significantly from the communities suspended in groundwater (Figure 3).

**Figure 4:**
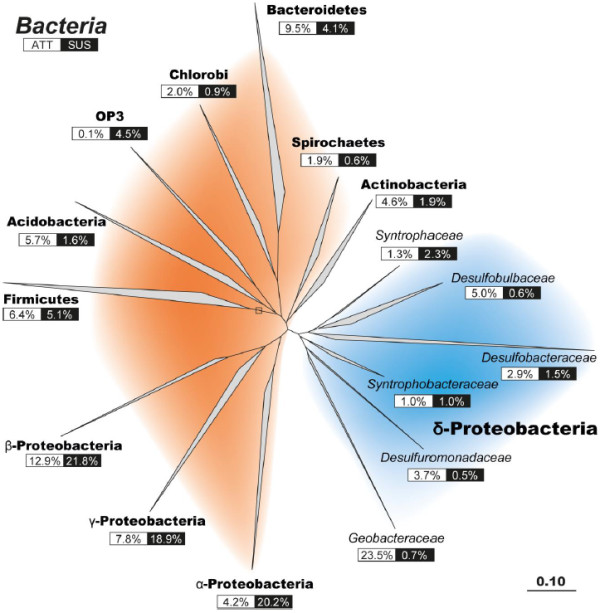
**Phylogenetic tree of bacterial 16S rRNA genes generated using sequences from the Greengenes database **[34]** and cloned sequences from this study.** The relative proportion of clones in the attached (ATT) or suspended (SUS) libraries is indicated below the label of each branch. Colored backgrounds distinguish the clades within the ∂-Proteobacteria (blue) from the other bacterial phyla (orange).

SIMPER analysis identified the OTUs that account for the differences in community assemblages. It showed that for bacteria, ATT communities differ from the SUS community largely because of several genera of ∆-Proteobacteria, primarily taxa associated with iron and sulfate reduction, that were more abundant in the fraction of cells that attached to our in situ samplers (Figure 4). Specifically, sequences classified as *Geobacter*, an iron-reducing genus, comprise 24% of the ATT community in a given well, but make up < 1% of sequences of the SUS community. Sequences most closely related to other iron-reducing phylotypes, *Desulfuromonadaceae* and *Geothrix*, were also more predominant in ATT communities than SUS, making up a combined 9% of attached cells compared to < 1% of suspended. Sulfate-reducing ∆-Proteobacteria within the families *Desulfobacteraceae* and *Desulfobulbaceae* were also more predominant in ATT samples than SUS. Sequences most closely related to these genera, on average, comprised 8% of the attached community but only 2% of the suspended. Conversely, members of the α-, β-, and γ-Proteobacteria were more predominant in the SUS fraction than the ATT (Figure 4). Sequences classified as belonging to *Burkholderiales*, *Sphingomonadaceae*, *Pseudomonadaceae*, and *Caulobacteraceae* represented 36% of SUS communities but only 5% of ATT communities. Sequences of other major bacterial phyla detected in the Mahomet, *Bacteroidetes* and *Firmicutes*, were of approximately equivalent abundance in attached and suspended fractions sampled from the aquifer.

**Figure 5:**
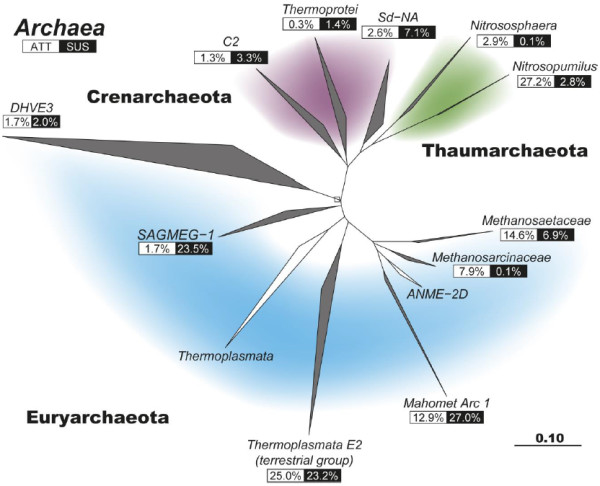
**Phylogenetic tree of archaeal 16S rRNA gene sequences generated using sequences from the Greengenes database (white branches) **[34]** and cloned sequences from this study (gray branches).** The relative proportion of clones in the attached (ATT) or suspended (SUS) libraries is indicated below the label of each branch. Colored regions highlight the different archaeal phyla: *Euryarchaeota* (blue), *Crenarchaeota* (purple), and *Thaumarchaeota* (green).

Among the archaea, SIMPER analysis revealed that sequences related to known methanogens and the phylum *Thaumarchaeota* differentiated the ATT community from the SUS community (Figure 5). Methanogens of families *Methanosarcinaceae* and *Methanosaetaceae* were three times as abundant in the attached fraction (23%) as in the suspended (7%), while *Thaumarchaeota* were nearly ten times more abundant in sediment samples (27%) as in groundwater (3%). Additionally, the SUS communities were distinguished from ATT communities by a greater relative abundance of sequences most closely related to the South African Gold Mine Euryarchaeal Group 1 (SAGMEG-1) and a novel group of archaea most closely related to the ANME-2D clade of anaerobic methane-oxidizers that we named “Mahomet Arc 1” (Figure 5). Mahomet Arc 1 sequences are most closely related to (>99% sequence identity) an archaeon linked to anaerobic methane oxidation in denitrifying bioreactors [46,47]. SAGMEG-1 sequences comprised 22% of SUS sequences yet only 2% of ATT sequences. Mahomet Arc 1 sequences were twice as abundant in groundwater as in sediment samples, composing 27% of the suspended fraction but only 13% of the attached. The abundance of the *Thermoplasmata* E2 group or any *Crenarchaeota* (clades C2, Sd-NA, and the *Thermoprotei*) did not vary appreciably between the attached and suspended fractions.

### Correlation of microbial community structure with groundwater chemistry

Because of the large difference between attached and suspended communities, each fraction was analyzed separately for evaluating how microbial community structure related to variations in groundwater chemistry. Among attached communities of bacteria and archaea, the chemical composition of groundwater appeared to be the key discriminant of community structure (Additional file 1: Figure S4). The structure of both bacterial and archaeal communities in NS wells, which contain negligible sulfate but high methane, differed significantly from communities identified from LS (low sulfate, low methane) and HS (high sulfate, negligible methane) wells (Table 3). However, bacterial and archaeal communities in LS and HS wells did not differ significantly. Furthermore, ANOSIM indicated that within the attached fraction the bacterial and archaeal communities, NS wells differed markedly from the LS and HS community wells, but there were an insufficient number of samples from the suspended fraction from NS wells sampled to determine whether or not these differences were statistically significant among the SUS communities (Table 3). Archaeal communities suspended in HS wells differed significantly from those suspended in LS wells, while bacterial communities in these same groups were not significantly different. MDS plots comparing attached communities of archaea and bacteria from HS and LS well areas of the aquifer formed overlapping clusters that were separate from communities in NS wells (Additional file 1: Figure S4). Similarly, MDS plots of the suspended communities in these wells show the one NS well where a SUS sample was available is plotted apart from the clusters of HS and LS wells (Additional file 1: Figure S5).

**Table 3:** **Results of analysis of similarity (ANOSIM)**^**a **^**between HS, LS, and NS wells**^**b**^

	***Bacteria***				***Archaea***			
	**ATT **^**c**^		**SUS **^**d**^		**ATT**		**SUS**	
	***R***_***ANOSIM***_	***p***	***R***_***ANOSIM***_	***p***	***R***_***ANOSIM***_	***p***	***R***_***ANOSIM***_	***p***
**HS - LS**	0.079	*11.9%*	0.019	*53.3%*	0.013	*51.7%*	0.493	*0.03%*
**HS - NS**	0.44	*0.02%*	–^e^	–^e^	0.857	*0.07%*	–^e^	–^e^
**LS - NS**	0.306	*0.08%*	–^e^	–^e^	0.599	*0.10%*	–^e^	–^e^

^a^*R*_*ANOSIM*_ ranges from a value of 0, which indicates communities in each group are identical, to 1, where communities in one group are completely distinct from the other. The value of *p* is the percentage chance that 10^6^ randomly generated groups produced a value of R_ANOSIM_ greater than the one given.

^b^ The concentration of sulfate in HS wells is > 0.2 mM, between 0.03 – 0.2 mM in LS wells, and less than 0.03 mM in NS wells.

^c^ ATT = Microbial communities attached to in situ samplers.

^d^ SUS = Microbial communities suspended in groundwater.

^e^ Insufficient NS samples for statistically valid ANOSIM.

**Figure 6:**
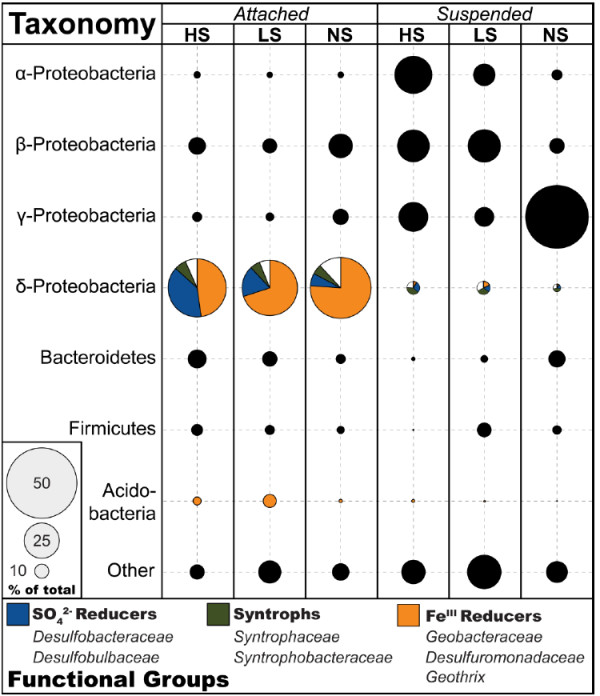
**The taxonomy and relative distribution of bacterial populations attached to the sediment of in situ samplers.** Sequences were classified to the genus level using Mothur [33] with the “Hugenholtz” taxonomic nomenclature in Greengenes [34]. The area of each circle is proportional to the percentage of sequences represented by that class within those wells, which are grouped together according to the concentration of sulfate in groundwater.

SIMPER analysis shows that the distribution of individual populations within the ∆-Proteobacteria drove the differentiation of the attached bacterial community structure among HS, LS, and NS wells. Sequences most closely related to iron-reducing (*Geobacter*) and sulfate-reducing (*Desulfobulbaceae* and *Desulfobacteraceae*) bacteria are relatively more abundant in LS and NS wells where sulfate concentrations were low (< 0.2 mM) compared to wells with higher sulfate concentrations (Figure 6). *Geobacter* sequences comprised 34% of all bacterial sequences in NS wells and 22% of LS wells, but only 15% of HS wells. Conversely, ∆-Proteobacteria clones related to families associated with sulfate reduction, *Desulfobulbaceae* and *Desulfobacteraceae*, were of lower relative abundance in bacterial communities in wells with low sulfate concentrations. In HS wells, members of these families represented 20% of all attached bacterial sequences, but comprised 8% of the total in LS wells and 3% in NS wells.

**Figure 7:**
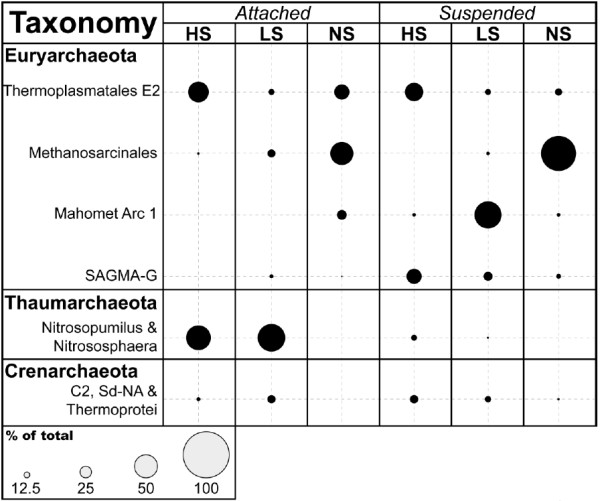
**The taxonomy and relative distribution of archaeal populations attached to the sediment of in situ samplers.** Sequences were classified to the genus level in Mothur [33] with the “Hugenholtz” taxonomic nomenclature in Greengenes [34]. The area of each circle is proportional to the percentage of sequences represented by that class within those wells, which are grouped together according to the concentration of sulfate in groundwater.

SIMPER analysis also shows that sequences classified as belonging to families of methanogens (*Methanosarcinaceae* and *Methanosaetaceae*) dominated the archaeal communities in both the suspended and attached fractions of NS wells, were considerably less abundant in LS wells, and were nearly absent in HS wells (Figure 7). In HS and LS wells, where few sequences in this group were detected, methane concentrations were low or undetectable (Figure 2). Clones from the *Methanosarcinales* comprise on average < 0.5% of the archaeal sequences in HS wells and 1 - 4% of the community in LS wells. In NS wells, which contain abundant methane, methanogen sequences represent 73 - 80% of the entire archaeal community. Euryarchaeal sequences from the Mahomet Arc 1, identified mostly in suspended communities, are more prevalent in LS wells (56%) relative to both HS and NS (~4% in each) wells (Figure 7).

## Discussion

The distinct physical and geochemical niches within the Mahomet aquifer harbour characteristic populations of bacteria and archaea. The microbial communities attached to the in situ sediment samplers (ATT) differed distinctly from those suspended in groundwater (SUS) (Figure 3), both in composition (Figures 4 &5) and, in the case of archaea, species richness (Additional file 1: Figure S2). While the number of OTUs we observed varied little between ATT and SUS bacteria and the two groups shared only one-third of their phylogenetic diversity, the archaeal community that colonized our in situ samplers was a distinct subset of the suspended community. Over 90% of ATT archaeal sequences were from OTUs that were also detected in the SUS fraction, yet 78% of SUS archaeal sequences were not detected in ATT samples (Table 2). This provides strong evidence that the most active and fastest-growing archaeal populations colonized the initially-sterile sediment contained in our in situ samplers. The phylogenetic distinction between ATT and SUS samples (Figure 3) provides further evidence that this is the case, because no such differentiation of ATT from SUS would be expected if the attachment of cells to the in situ samplers was driven purely by neutral factors such as random adhesion rather than selective colonization [15,48].

Sequences related to iron-reducing and sulfate-reducing bacteria are much more predominant among the ATT communities when compared to their corresponding SUS communities (Figure 6). Geochemical evidence also supports concurrent iron reduction and sulfate reduction processes in this area of the Mahomet aquifer [17,22]. The near-absence of these functional populations from SUS groundwater samples suggests that their niche is likely localized to the surface of mineral grains. This makes sense since available ferric iron was associated with the sediment sand used in the traps. This result is not surprising in the case of iron reducers, due to the highly insoluble nature of ferric iron minerals expected in the Mahomet (pH = 7.1–7.9). Iron reducers such as *Geobacter* require some mechanism of physical attachment to ferric minerals in order to respire [49]. Sulfate, conversely, is highly soluble, meaning sulfate reducers do not necessarily require attachment to aquifer sediment in order to respire. The greater abundance of apparent sulfate-reducing bacteria in ATT samples relative to SUS may occur because these organisms benefit from proximity to iron reducers, whose generation of ferrous iron prevents toxic sulfide from accumulating in solution [2,42]. When ferrous iron and sulfide are produced simultaneously, they precipitate as the minerals mackinawite (FeS) and greigite (Fe_3_S_4_) [50], limiting the buildup of both reaction products in groundwater and maintaining the thermodynamic drive for each group’s metabolism [51]. Iron reducers have also appeared to benefit from the presence of active sulfate reduction perhaps for the same reason [42]. The predominance of sulfate reducers along with iron reducers in aquifer sediment over groundwater suggests that the two groups may benefit from concurrent respiration.

This simultaneous reduction of both iron and sulfate exemplifies mutualistic behavior, which exists when two distinct groups of organisms benefit from each other’s activity [52]. So despite sulfate reducers and iron reducers competing for the same electron donors in the Mahomet aquifer, by working together they prevent product inhibition. Therefore, rather than being excluded due to thermodynamic constraints by iron reducers as is often suggested [19,20], sulfate reducers seem to be thriving alongside them in the Mahomet aquifer. The relative richness of iron-reducing bacteria as a proportion of total OTUs only exceeded that of sulfate reducers when sulfate concentrations were below 0.2 mM. Although the relative abundance of an OTU does not necessarily correlate with the cell numbers of a particular functional group, the data do suggest that both metabolisms are maintained in the presence of sulfate. What appears to change is the relative proportion of each functional group as the sulfate concentration changes. Indeed, the primary discriminant of microbial community structure in the Mahomet was the concentration of sulfate in groundwater as indicated by ANOSIM (Table 3) and MDS analyses (Additional file 1: Figures S4 and S5). This is in agreement with results from recent studies which suggest that in the presence of sulfate-reducing bacteria, iron reducers will modify their rate of respiration in order to effectively remove sulfide to the benefit of both groups [42].

The availability of sulfate also appeared to control archaeal community structure within the Mahomet aquifer. MDS plots comparing archaeal community structure across the aquifer show a distinct clustering of wells with similar amounts of sulfate in the groundwater (Additional file 1: Figures S4 and S5). This differentiation is largely driven by differences in the relative abundance of methanogens compared to other archaea under high and low sulfate conditions. SIMPER analysis showed methanogen-like taxa to comprise a lower proportion of the total archaea in wells where the concentration of sulfate was > 0.03 mM (HS and LS wells), but the same sequences made up nearly 80% of all those obtained from NS wells (Figure 7). These results were commensurate with the concentration of methane detected in groundwater, which was nearly two orders of magnitude higher in NS wells than in HS or LS wells (Figure 2).

The relative abundance of methanogen 16S rRNA gene sequences correlates well with the inverse relationship between sulfate and methane concentrations that was observed in the wells sampled. This has also been observed in other aquifers, where it has been interpreted as a result of sulfate-reducing bacteria outcompeting methanogens and maintaining concentrations of H_2_ too low for the latter to respire [53,54]. Assuming a minimum of 10 kJ mol^-1^ (∆*G*_min_) is required for these microorganisms to gain energy via respiration [55], our thermodynamic calculations show that sufficient energy exists for both methanogens and sulfate reducers to respire in every well sampled. In addition, despite the dynamic range of methane and sulfate concentrations shown in Figure 1, H_2_ concentrations show no correlation to the relative abundance of sulfate reducers or methanogens as would be expected if thermodynamics controlled which type of metabolism could occur [53,56]. The very low relative abundance of methanogens in HS and LS wells can instead be explained by the kinetics, rather than the thermodynamics, of microbial metabolism. Methanogenesis provides organisms less energy per mole of substrate consumed than sulfate reduction, and kinetic theory suggests methanogens are not able to respire quickly enough to maintain a viable population in the presence of active sulfate reduction [2,57]. Laboratory studies of co-cultured methanogens and sulfate reducers indicate that methanogenesis ceases following the addition of sulfate to an active biofilm [58]. Even after switching back to a sulfate-free medium, the biofilm required two months to reach its previous level of activity, suggesting the methanogens had died off rather than simply being inhibited by sulfate. The relative low abundance of sulfate reducers observed in NS wells (Figure 6) despite sufficient available energy (Additional file 1: Table S1), conversely, provides further evidence that thermodynamics is not necessarily the ultimate control on the distribution of microbial activity. Rather, because sulfate enters the Mahomet aquifer mainly via leakage from the bedrock in a limited area of east-central Illinois [17], the flux of sulfate into NS areas of the Mahomet aquifer is likely too low to support a stable population of sulfate reducers.

In addition to controlling the abundance of methanogens, the concentration of sulfate also controls the abundance of Mahomet Arc 1 sequences, a group most closely related to the clade ANME-2D (Figure 5). Specifically Mahomet Arc 1 sequences match most closely archaea shown to anaerobically oxidize methane (AOM) [46,47]. In this aquifer system, Mahomet Arc 1 archaea are present in nearly every well and were the most abundant member of the archaeal community in LS wells (Figure 7). Archaea in the ANME-2D clade have been implicated as the methane-oxidizing, hydrogen-producing half of a syntrophic partnership that works in tandem with hydrogen-consuming microbes such as sulfate reducers or denitrifiers [59]. These hydrogenotrophs keep H_2_ concentrations low enough to allow anaerobic methane oxidation to remain thermodynamically favorable for the ANME organisms [55]. Mahomet Arc 1 sequences are 99% similar to those found in an ecosystem confirmed to be anaerobically oxidizing methane [46], therefore it appears reasonable to hypothesize that this group is also serving this function in the Mahomet.

Despite the abundance of Mahomet Arc 1 sequences in our LS well samples, AOM via reverse methanogenesis remains endergonic at the bulk concentration of H_2_ measured in Mahomet groundwater (Additional file 1: Table S1). If methane oxidation occurs in the Mahomet, it must happen within aggregates or biofilms which allow a lower local concentration of H_2_ to be maintained by a hydrogen-consuming partner. AOM becomes energetically favorable in LS wells at concentrations of H_2_(aq) of less than roughly 0.2 nM (Additional file 1: Figure S3), which is 1–2 orders of magnitude less than the bulk concentration of H_2_ in groundwater. Depending upon the kinetics of H_2_ consumption, such a gradient would be feasible inside a biofilm [55]. Alternatively, recent studies have demonstrated direct electron transfer between cells without the intermediate formation of H_2_[60,61]. If this occurs close cell contact would still be required for AOM to be feasible. Our study, however does not resolve whether such specific close cell associations occur in the Mahomet aquifer or whether these are specifically associated with AOM in this system. We hope to address this more fundamentally in a future study.

The discovery of Mahomet Arc 1, which appears to be associated with AOM, in a pristine aquifer suggests the anaerobic oxidation of methane may be an additional important metabolic pathway in this system. The heterogeneity of aquifer sediments also leads to numerous microenvironments whose redox chemistry can differ greatly from the bulk groundwater [62]. Molecular diffusion and advective transport can transport methane from the highly reduced zones where it is produced into areas where it might be consumed through an AOM-mediating syntrophic partnership. Because the rates at which CH_4_ is produced and potentially consumed are difficult to quantify in situ, anaerobic methane oxidation is frequently overlooked in groundwater ecosystems [10]. The abundance of Mahomet Arc 1 sequences and their correlation to the concentration of sulfate then not only suggests the potential importance of AOM as a biogeochemical pathway in the Mahomet, but underscores the largely-untapped potential provided by molecular microbial ecology to better define redox processes in pristine aquifers.

## Conclusions

While this study greatly increases our understanding of the microbial communities that catalyze the biogeochemical cycling of carbon and metals in the Mahomet aquifer, additional studies are needed to shed light on the dynamics of microbial activities of this and other subsurface systems over time. Moreover, molecular surveys represent an important foundation for studies trying to understand how changes in subsurface chemistry may impact subsurface communities exposed to anthropogenic perturbations such as geological carbon sequestration and hydrologic fracturing of gas-rich strata, both of which may lead to changes in groundwater flows and chemistries. Further, our results indicate that appropriate monitoring schemes must consider the assessment of the microbial fraction associated with subsurface biofilm communities as their composition and activities might not be easily predicted by targeting the suspended fraction extracted with groundwater.

## Abbreviations

OTU: Operational taxonomic unit; MDS: (nonmetric) Multidimensional scaling; ANOSIM: Analysis of similarity; SIMPER: Similarity percentage; AOM: Anaerobic oxidation of methane.

## Competing interests

The authors declare no competing interests.

## Authors’ contributions

TMF, RAS, and CMB conceived of the study, planned, and executed the sampling of Mahomet aquifer wells. TMF extracted DNA, performed geochemical analyses, aligned sequence data, performed phylogenetic and statistical analyses, and calculated the energy available for microbial respiration. HR and JWSD carried out the sequencing reactions. TMF, RAS, and JWSD reviewed and analyzed the phylogenetic and statistical data. TMF, RAS, CMB, NJA, and JWSD drafted the original manuscript and all authors provided critical revisions of the manuscript text and figures. All authors read and approved the final manuscript.

## Supplementary Material

Additional file 1: Table S1 Energy available for microbial respiration. **Figure S1.** Collectors curves showing how the total richness of the bacterial community increases with greater sampling depth. **Figure S2.** Collectors curves showing how the total richness of the archaeal community increases with greater sampling depth. **Figure S3.** Available energy (∆*G*_A_) for either the anaerobic oxidation of methane (AOM) or methanogenesis with increasing amounts of dihydrogen (H_2_) in Mahomet aquifer groundwater. **Figure S4.** Multidimensional scaling (MDS) ordination of the Bray-Curtis coefficients of similarity for attached microbial communities in the Mahomet aquifer. **Figure S5.** Multidimensional scaling (MDS) ordination of the Bray-Curtis coefficients of similarity for suspended microbial communities in the Mahomet aquifer. Click here for file
