# Supplementary material for: Functional microbial diversity explains groundwater chemistry in a pristine aquifer
Source: BMC Microbiol. 2013 Jun 24;13:146. doi: 10.1186/1471-2180-13-146 (PMC3700874; doi:10.1186/1471-2180-13-146)
Supplement: Additional file 1: Table S1 — Energy available for microbial respiration. Figure S1. Collectors curves showing how the total richness of the bacterial community increases with greater sampling depth. Figure S2. Collectors curves showing how the total richness of the archaeal community increases with greater sampling depth. Figure S3. Available energy (∆GA) for either the anaerobic oxidation of methane (AOM) or methanogenesis with increasing amounts of dihydrogen (H2) in Mahomet aquifer groundwater. Figure S4. Multidimensional scaling (MDS) ordination of the Bray-Curtis coefficients of similarity for attached microbial communities in the Mahomet aquifer. Figure S5. Multidimensional scaling (MDS) ordination of the Bray-Curtis coefficients of similarity for suspended microbial communities in the Mahomet aquifer. [file 1471-2180-13-146-S1.docx]

**Functional microbial diversity explains groundwater chemistry in a pristine aquifer**

**Theodore M. Flynn^1,2^, Robert A. Sanford^2^, Hodon Ryu^3^, Craig M. Bethke^2^, Audrey D. Levine^3,4^, Nicholas J. Ashbolt^3^, and Jorge W. Santo Domingo^3§^**

^1^Biosciences Division, Argonne National Laboratory, Argonne, IL 60439, USA

^2^Department of Geology, University of Illinois at Urbana-Champaign, Urbana, IL 60616, USA

^3^Office of Research and Development, U.S. Environmental Protection Agency, Cincinnati, OH 45248, USA

^4^Battelle, Washington, DC 20024, USA

^§^Corresponding author

Supplemental information

Material and Methods

**In situ microbial samplers.** In situ samplers were used to recover attached microorganisms from the groundwater as previously described [1, 2]. These microbial “traps” were composed of autoclaved Mahomet aquifer sand obtained during the drilling of a private well in the Banner formation. The amount of acid-extractable ferric iron in the sand was measured to be 7.3 (±0.9) µmol g^–1^ (dry weight) using the ferrozine method following reduction of the extracted ferric iron using 6.25 N hydroxylamine [3]. Storage containers were filled completely with sand and groundwater to minimize contact with the atmosphere, and transported to the laboratory on ice coolers within four hours of collection. Upon return to the lab, the sediment was immediately washed to remove drilling mud and to minimize the re-oxidation of any ferrous iron sorbed to mineral surfaces. The washed sediment was then autoclaved, dried, and loaded into AquaClear 20-mesh bags (CD-120490, Doctors Foster and Smith™, Rhinelander, WI) for deployment. Traps were placed into monitoring wells for at least three months, allowing diverse bacterial communities to colonize sterile sediments and reach steady state populations.

**Nucleic acid extraction and PCR conditions.** Nucleic acid was extracted using the method of Tsai and Olson [4] with some modifications. Cells were lysed by incubating individual filters or 4 g of sediment in 2 mL of the lysis solution at 37°C for 30 min. Detergent solution (2 mL; 0.1 M NaCl, 0.5 M Tris-Cl, 10% sodium dodecyl sulfate, pH = 8.0) was then added and incubated for 30 min, after which the samples were subjected to three freeze-thaw cycles using liquid nitrogen and a 55°C water bath. Proteins were removed from the aqueous phase using equal volumes of phenol (pH = 7.8), phenol:chloroform:isoamyl alcohol (25:24:1, pH = 7.8) and chloroform:isoamyl alcohol (24:1). DNA was precipitated overnight by adding an equal volume of isopropanol followed by 10.5 M ammonium acetate to a final concentration of 2.5 M. The nucleic acid precipitate was centrifuged at 9,000 × *g* for 30 min at 4°C, washed in 70% molecular grade ethanol, and resuspended in Tris-EDTA buffer (pH = 8.0). The amount and quality of DNA recovered was estimated using agarose gel electrophoresis. Negative controls consisted of unused filters and sediment traps.

Bacterial primers 8F (5'-AGAGTTTGATCCTGGCTCAG-3') and 787R (5'-GGACTACCAGGGTATCTAAT-3') and archaeal primers 25F (5'-CYGGTTGATCCTGCCRG-3') and 958R (5'-YCCGGCGTTGAMTCCAATT-3') were used to amplify 16S rRNA genes [5, 6]. Amplification reactions contained 5 U of Ex Taq™ DNA polymerase (Takara Bio USA, Madison, WI), 5 µL of 10X concentrated Ex Taq™ Buffer, 4 µL of a 2.5 mM mixture of dNTPs, 3 µL each of forward and reverse primers (2.0 µM stock concentration), and 2 µL of template DNA (50 µl total volume). Amplification conditions for the bacterial assay included an initial denaturation step of 4 min at 94°C, followed by 35 cycles of 30 s at 94°C, 30s at 56°C, and 1 min at 72°C, with a final extension step of 7 min at 72°C. For Archaea, the reaction began with an initial denaturation step (4 min at 94°C), followed by 35 cycles of 90 s at 94°C, 90 s at 58°C, and 2 min at 72°C, with a final extension step of 12 min at 72°C. Amplification products were screened using gel electrophoresis and GelStar™ dye (BioWhittaker Molecular Applications, Rockland, ME).

# References

1. Flynn TM, Sanford RA, Bethke CM: **Attached and suspended microbial communities in a pristine confined aquifer.** *Water Resour Res* 2008, **44:**W07425.

2. Flynn TM, Sanford RA, Santo Domingo JW, Ashbolt NJ, Levine AD, Bethke CM: **The active bacterial community in a pristine confined aquifer.** *Water Resour Res* 2012, **48:**W09510.

3. Sørensen J: **Reduction of Ferric Iron in Anaerobic, Marine Sediment and Interaction with Reduction of Nitrate and Sulfate.** *Appl Environ Microbiol* 1982, **43:**319-324.

4. Tsai YL, Olson BH: **Rapid method for direct extraction of DNA from soil and sediments.** *Appl Environ Microbiol* 1991, **57:**1070-1074.

5. Elshahed MS, Najar FZ, Roe BA, Oren A, Dewers TA, Krumholz LR: **Survey of archaeal diversity reveals an abundance of halophilic *Archaea* in a low-salt, sulfide- and sulfur-rich spring.** *Appl Environ Microbiol* 2004, **70:**2230-2239.

6. Buchholz-Cleven B, Rattunde B, Straub K: **Screening for genetic diversity of isolates of anaerobic Fe(II)-oxidizing bacteria using DGGE and whole-cell hybridization.** *Syst Appl Microbiol* 1997, **20:**301-309.

7. Schloss PD, Westcott SL, Ryabin T, Hall JR, Hartmann M, Hollister EB, Lesniewski RA, Oakley BB, Parks DH, Robinson CJ, et al: **Introducing mothur: open-source, platform-independent, community-supported software for describing and comparing microbial communities.** *Appl Environ Microbiol* 2009, **75:**7537-7541.

8. Alperin MJ, Hoehler TM: **Anaerobic methane oxidation by archaea/sulfate-reducing bacteria aggregates: 1. Thermodynamic and physical constraints.** *Am J Sci* 2009, **309:**869-957.

9. Schink B: **Energetics of syntrophic cooperation in methanogenic degradation.** *Microbiol Mol Biol Rev* 1997, **61:**262-280.

# Supplemental Tables

## Table S1 - Energy Available for Microbial Respiration

Average energy available (Δ*G*_A_) for sulfate reduction, methanogenesis, and anaerobic methane oxidation in wells in the Mahomet aquifer where the concentration of sulfate is high (HS > 0.2 mM), low (LS = 0.03 – 0.2 mM), or negligible (NS < 0.03 mM). The average ∆G_A_  for the coupled oxidation of methane to sulfate reduction without H2 generation is also shown. Concentrations of other reactants were obtained from the average observed in the wells sampled. Negative ∆G_A_ values indicate the designated metabolism is not energetically favorable under the given conditions.

|  | Δ*G*_A_ (kJ mol^−1^) | | |
| --- | --- | --- | --- |
| *Anaerobic Methane Oxidation* | \| **HS** \| **LS** \| **NS** \| \| --- \| --- \| --- \| | | |
| $\mathrm{CH}_{4}\text{(aq) + }{3H}_{2}O\to\mathrm{HCO}_{3}^{-}+H^{+}+4H_{2}(\mathrm{aq})$ | −40±15 | −32±13 | −37±8 |
|  |  |  |  |
| *Sulfate Reduction* |  | | |
| $\mathrm{SO}_{4}^{2-}+4H_{2}(\mathrm{aq})+H^{+}\to\mathrm{HS}^{-}+4H_{2}O$ | 74±20 | 62±14 | 72±9 |
|  |  |  |  |
| *Methanogenesis* |  | | |
| $\mathrm{HCO}_{3}^{-}+H^{+}+4H_{2}(\mathrm{aq})\to\mathrm{CH}_{4}\text{ + }{3H}_{2}O$ | 40±15 | 32±13 | 37±8 |
|  |  |  |  |
| *Anaerobic Methane Oxidation coupled to Sulfate Reduction* |  |  |  |
| $\mathrm{CH}_{4}\text{(aq) + }{\mathrm{SO}_{4}^{2-}}\to\mathrm{HCO}_{3}^{-}+\mathrm{HS}^{-}+H_{2}O$ | 34 | 30 | 35 |

# Supplemental Figures

**
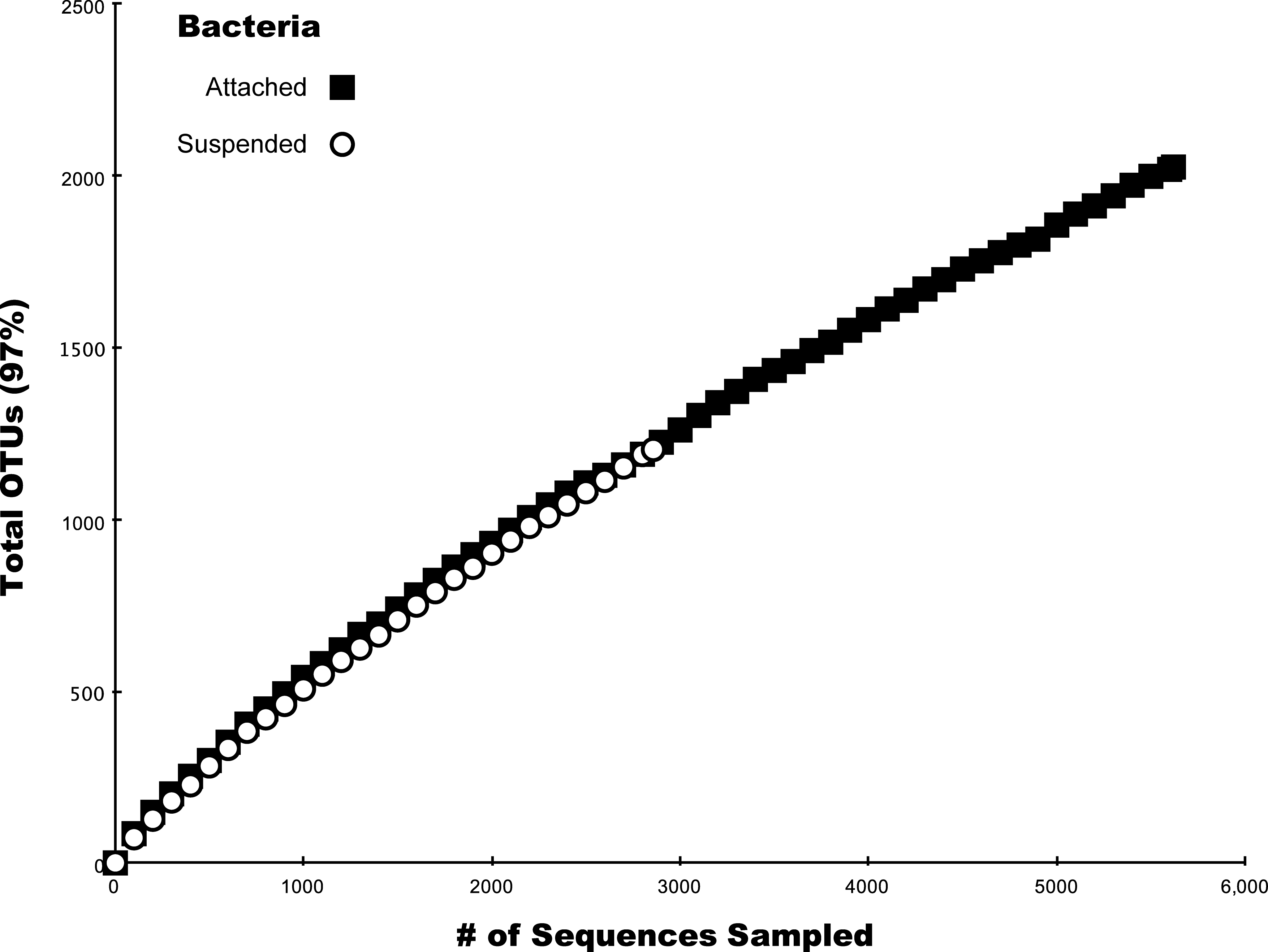
**

Figure S1. Collectors curves showing how the total richness of the bacterial community increases with greater sampling depth. OTUs in this plot were created using an average sequence similarity cutoff of 97% in Mothur [7].


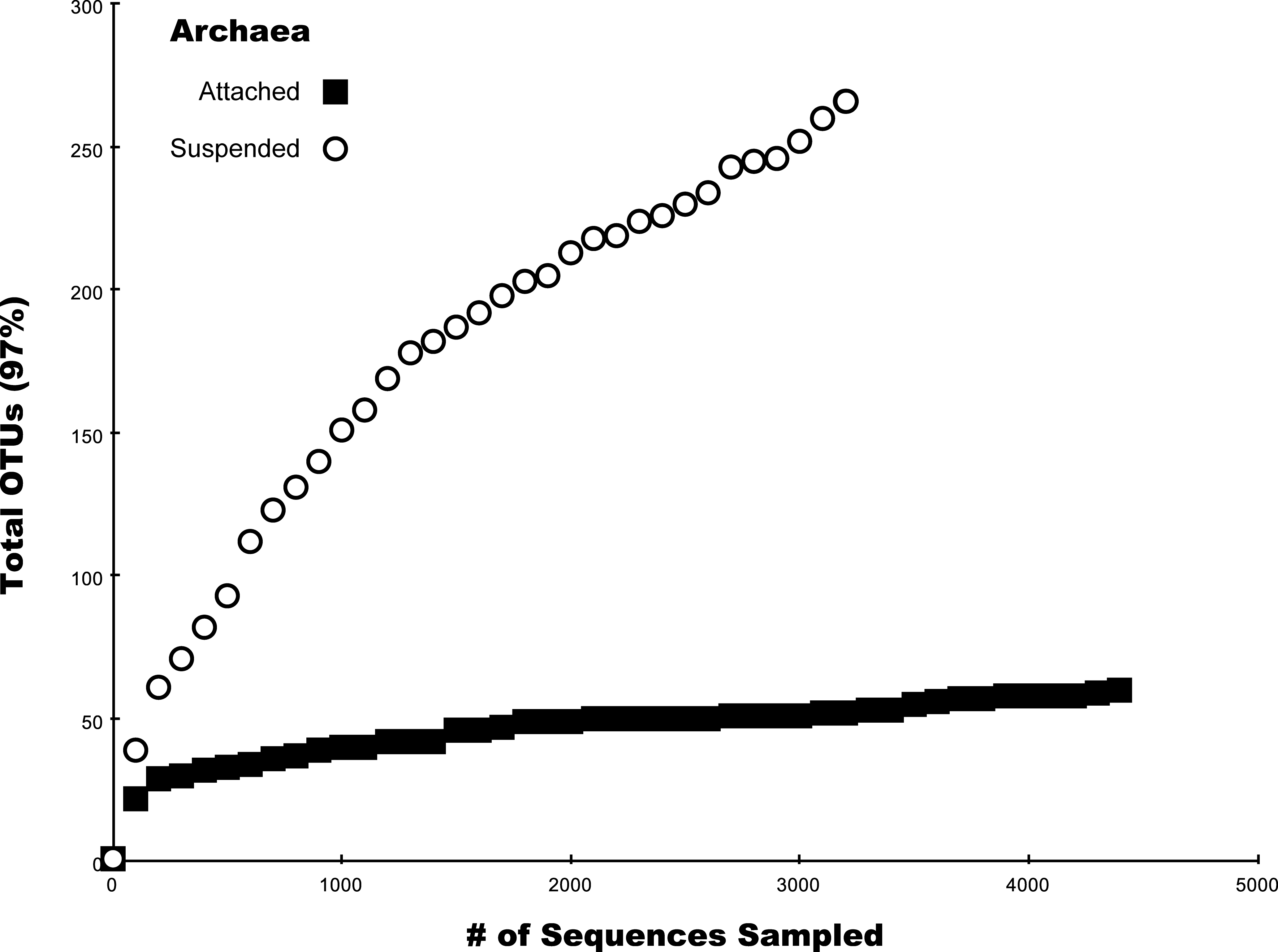


Figure S2. Collectors curves showing how the total richness of the archaeal community increases with greater sampling depth. OTUs in this plot were created using an average sequence similarity cutoff of 97% in Mothur [7].


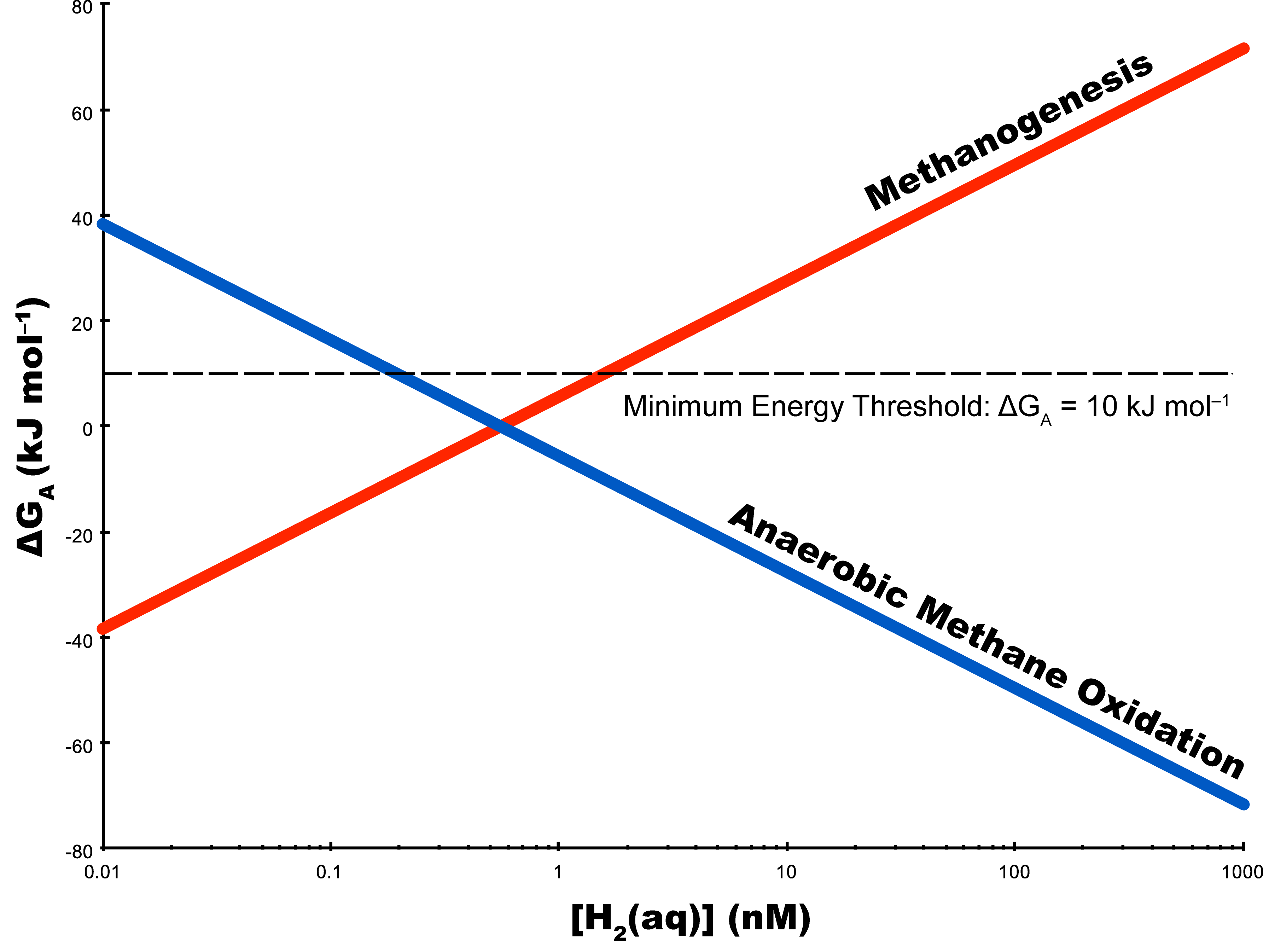


Figure S3. Available energy (Δ*G*_A_) for either the anaerobic oxidation of methane (AOM) or methanogenesis with increasing amounts of dihydrogen (H_2_) in Mahomet aquifer groundwater^[[1]](#footnote-1)^. The segment of each line that lies above the dotted indicates where the energy yield from that particular redox process is sufficient to support microbial growth (Δ*G*_min_). Δ*G*_min_ is estimated to be approximately 10 kJ mol^−1^ [8, 9].


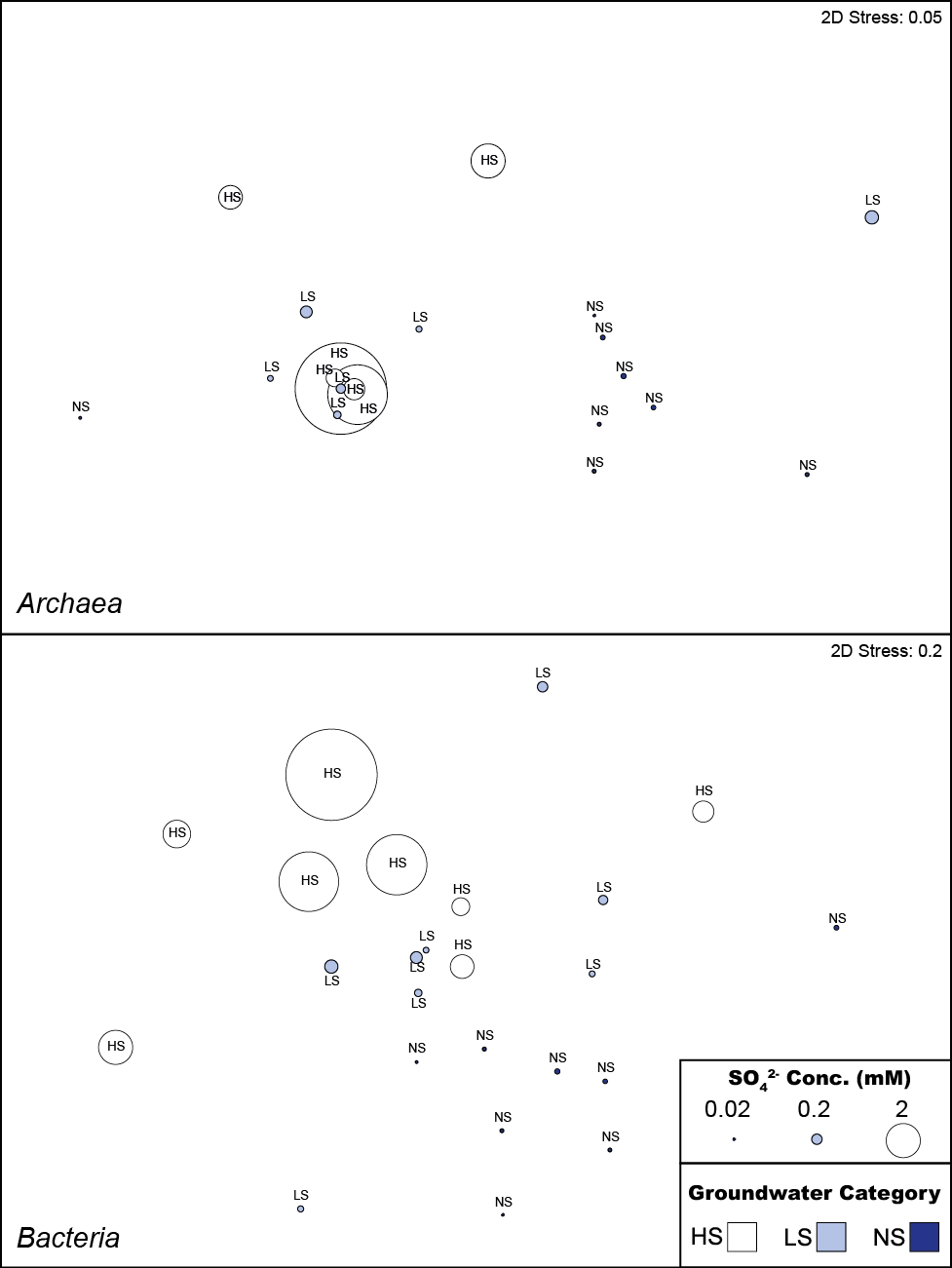


Figure S4. Multidimensional scaling (MDS) ordination of the Bray-Curtis coefficients of similarity for attached microbial communities in the Mahomet aquifer. The stress indicated in the upper right corner is the amount of strain imposed on the ordination when fitting it into two dimensions. The size of the circle representing a particular community is proportional to the concentration of sulfate in groundwater from the well where that sample was taken.


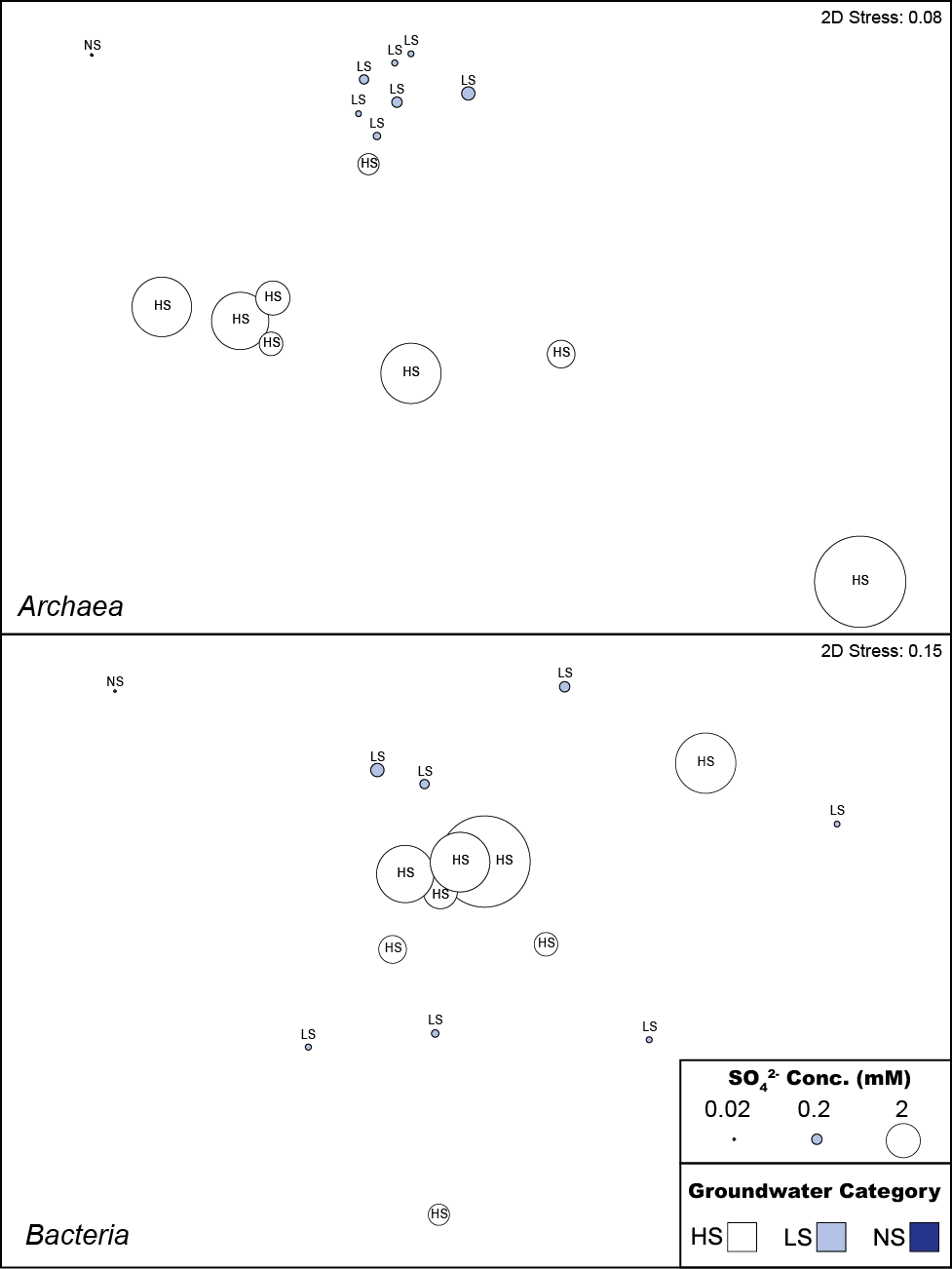


Figure S5. Multidimensional scaling (MDS) ordination of the Bray-Curtis coefficients of similarity for suspended microbial communities in the Mahomet aquifer. The stress indicated in the upper right corner is the amount of strain imposed on the ordination when fitting it into two dimensions. The size of the circle representing a particular community is proportional to the concentration of sulfate in groundwater from the well where that sample was taken.

1. The values used to make these calculations, apart from H_2_, are average values for LS wells in the Mahomet aquifer (T = 14ºC; pH = 7.4, [HCO_3_^−^] = 7.6 mM; [CH_4_(aq)] = 15 µM, total ionic strength = 33 mM. [↑](#footnote-ref-1)
